# 

**Published:** 1892-12

**Authors:** 


### CONTENTS.

ORIGINAL COMMUNICATIONS—

Compatibility of Conservative and Aggressive Surgery. By J. McFadden Gaston, M. D., Atlanta, Ga. .... 577

The Treatment of Salpingitis by Depletion and Drainage, Secured by Electricity. By Augustin H. Goelet, M. D., New York .... 586

A Case of Ovarian Pregnancy—Laparotomy—Cure. By Emory Lanphear, M. D., Ph. D., Kansas City, Mo. .... 590

The Shurly-Gibbes Treatment of Tuberculosis. By H. Longstreet Taylor, M. D., A. M., Asheville, N. C. .... 593

SOCIETY REPORT—

Southern Surgical and Gynæcological Association .... 594

CORRESPONDENCE—

From New York .... 605

EDITORIAL—

A Bill for a Medical Examining Board .... 611

The Southern Medical College Association .... 615

The "Social Evil" .... 617

SELECTIONS .... 620

MEDICAL ITEMS .... 633

BOOK REVIEWS .... 636

INDEX TO ADVERTISEMENTS .... xii

ITEMS OF INTEREST .... x-xi

